# Cross-Frequency Dependence Overlap in the Chinese A-Share Dependence Network

**DOI:** 10.3390/e28091038

**Published:** 2026-09-21

**Authors:** Jimao Mo, Jiaqi Zhang, Shijia Chen, Hua Zhang

**Affiliations:** 1 College of Engineering Physics, Shenzhen Technology University, Shenzhen 518118, China; 202300803086@stumail.sztu.edu.cn; 2 College of Artificial Intelligence, Shenzhen Technology University, Shenzhen 518118, China; 202401103050@stumail.sztu.edu.cn; 3 Shenzhen Key Laboratory of Ultraintense Laser and Advanced Material Technology, Center for Intense Laser Application Technology, Shenzhen Technology University, Shenzhen 518118, China; 4 College of Applied Sciences, Shenzhen University, Shenzhen 518060, China

**Keywords:** wavelet multiresolution analysis, community detection, dual-channel spectral clustering, financial networks, Chinese A-share market, cross-frequency dependence overlap

## Abstract

Multiresolution analysis is widely applied to equity markets on the assumption that different frequency bands capture distinct trading behaviors and information flows. Whether those bands reveal structurally distinct equity communities, or mostly re-express a shared dependence backbone, remains unresolved. We address this question in the Chinese A-share market using the maximal overlap discrete wavelet transform (MODWT) and a dual-channel affinity construction that processes positive and negative similarities separately before fusing them into a nonnegative matrix for normalized spectral clustering, over a grid of 15,360 configurations spanning wavelet bases, decomposition levels, and time windows. In the reference configuration (K=25), the adjusted Rand index (ARI) against the Shenwan first-level industry classification is 0.381, comparable to the strongest correlation-based baseline and with no singleton communities. Best-over-grid ARI declines monotonically from 0.381 at decomposition level 2 to 0.139 at level 6, in every time window and across the full configuration ensemble. Band-wise stock-pair similarity vectors show substantial rank agreement (mean Spearman ρ = 0.769), yet cross-band normalized mutual information averages 0.123 over 64 equal-frequency bins, and independently clustered bands give distinct partitions (mean pairwise ARI = 0.328). Rank agreement falls to 0.677 after market removal and to 0.486 after sequential market-and-industry removal. These results document measurable but incomplete cross-frequency dependence overlap, alongside declining industry alignment with increasing decomposition depth within the tested pipeline.

## 1. Introduction

Financial markets are complex adaptive systems [1,2]. Asset price fluctuations are shaped jointly by heterogeneous agents, macroeconomic cycles, policy shocks, industry linkages, investor sentiment, and micro-level trading behavior. Understanding the dependence structure among assets is therefore important for econophysics, systemic-risk analysis, and portfolio construction [3]. Complex-network methods provide a natural language for this problem. Mantegna [4] introduced the minimum-spanning-tree approach for extracting hierarchical market organization from return correlations, while Tumminello et al. [5] proposed the planar maximally filtered graph as a richer filtered representation. Mantegna and Stanley [3] provide the broader statistical-physics foundation for studying financial-market correlations and scaling behavior. Subsequent studies show that market networks become more compact and centralized during crises [6], that tail-dependence networks can differ from normal-period dependence structures [7], and that community-detection tools applied to Chinese stock data can recover economically meaningful intra- and inter-community relationships [8].

The Chinese A-share market provides a useful empirical setting for this problem. It contains more than five thousand listed stocks, spans a wide range of industries, and has institutional features such as strong retail participation, active regulatory involvement, and daily price limits [9,10]. These features make its dependence structure potentially different from that of mature markets and motivate a large-scale diagnostic study of whether different time scales reveal distinct market organization. Recent Chinese-market network research has moved beyond static maps toward dynamic market structure. Huang et al. [11], for example, construct dynamic partial-correlation networks for SSE 180 stocks and use a graph-neural community-detection framework to trace market phases, node migration, and community attractiveness. Their study shows how stock-network modularity and migration behavior can be used to interpret market regimes and stock behavior. The present paper is complementary: it studies a much larger A-share universe and examines the extent of dependence overlap across wavelet frequency bands, the agreement between band-specific community partitions, and the association between decomposition depth and industry alignment.

The time-scale dimension remains difficult to interpret. The heterogeneous market hypothesis [12,13] suggests that investors with different horizons may respond to different frequencies of information. Related frequency-domain connectedness research also shows that financial connectedness can vary across short-, medium-, and long-term cycles [14]. Wavelet analysis is a standard tool for nonstationary financial time series: Mallat [15] establishes the multiresolution decomposition framework, in which a signal is recursively split into low-frequency approximation coefficients and high-frequency detail coefficients. In finance, wavelets have been used for volatility decomposition, risk transmission, forecasting, market co-movement, and pairwise coherence, but their implications for community detection require distinguishing dependence overlap across frequency bands from agreement between community partitions and alignment with an external industry taxonomy.

Several wavelet-based financial studies are particularly related. MODWT is widely used to decompose financial return series [16], preserving time alignment across scales without decimation, although finite-sample boundary effects still require explicit treatment. Wang, Xie, and Chen [17] combine MODWT with MST and PMFG to construct multiscale correlation networks for U.S. stocks and examine edge persistence across scales. Rua and Nunes [18] use wavelets to study market risk in emerging markets, and Gençay et al. [19] analyze information flows across investment horizons. Li et al. [20] combine wavelet decomposition with forecasting models, while Lahmiri [21] uses wavelet denoising before clustering financial assets. These studies show that wavelets are economically meaningful, but most use them for volatility decomposition, risk analysis, pairwise coherence, forecasting, or separate network construction at different scales. These applications motivate a joint assessment of cross-band stock-pair dependence, agreement between band-specific community partitions, and the sensitivity of industry alignment to cross-scale aggregation in a large stock network.

This paper uses a WaveClust-style multiresolution framework [22] to test that premise. WaveClust was proposed for high-resolution ecological time series, where communities should be supported by associations that appear across multiple temporal scales. In ecological systems, this premise is plausible because tides, seasonal cycles, annual succession, and other physical processes may generate distinct frequency-specific community patterns. Transferring the idea to finance requires testing rather than assuming the same cross-scale heterogeneity, because macroeconomic shocks, sectoral exposure, and liquidity effects may generate similar dependence patterns across time resolutions. Random-matrix analysis distinguishes a market-wide mode and sector-related modes from a largely random eigenvalue bulk [23], while factor-removal experiments show that common factors materially affect stock-network connectivity and portfolio diversification [24]. Liquidity co-moves with market- and industry-wide liquidity [25], and common order-flow factors explain a substantial part of return commonality [26]. These findings motivate a common-factor interpretation but do not by themselves establish dependence overlap across wavelet bands. This raises two related questions: how much do stock-pair dependence representations and community partitions overlap across wavelet frequency bands, and how does industry alignment change with decomposition depth? Addressing these questions helps assess the empirical behavior of a WaveClust-style framework in financial markets. The results may be consistent with denoising and common-factor interpretations, but neither mechanism is established by cross-band agreement or changes in industry alignment alone.

To address these questions, the WaveClust framework serves as a diagnostic apparatus: it examines cross-band agreement in stock-pair similarity rankings, evaluates sensitivity to the layer-distance weighting parameter *k*, and measures community alignment with the Shenwan industry classification using the adjusted Rand index (ARI). Industry labels do not enter the construction of individual partitions, but they inform the candidate community counts and the ARI-based comparison and selection of configurations. The preprocessing follows log-return transformation, winsorization, and stock-wise standardization on actively traded stocks, with MODWT providing shift-invariant decomposition. Evidence of positive within-sector and negative between-sector correlations in the sector mode motivates considering both signs of co-movement [27]. The method combines WaveClust-style multiresolution feature construction with a dual-power affinity score that processes positive and negative similarities in separate channels before combining them into a nonnegative matrix for spectral clustering. The resulting affinity reflects both channels but does not retain an explicit or recoverable edge sign. A grid search over 15,360 configurations—spanning four wavelet bases, five decomposition levels, four time windows, and multiple clustering hyperparameters—systematically evaluates sensitivity to these choices. The analysis focuses on cross-frequency agreement in dependence rankings and changes in industry alignment across decomposition levels. These are distinct quantities: lower ARI against Shenwan labels does not exclude economically meaningful community structure organized along other dimensions [28].

This paper makes two contributions. First, it documents declining industry alignment with increasing wavelet decomposition level in the aggregate results of a 15,360-configuration grid search and separately identifies substantial cross-frequency agreement in stock-pair dependence rankings in the Chinese A-share market. The mean cross-band Spearman correlation of ρ=0.769 indicates similar rankings of stock-pair similarities across the analyzed bands. We describe this as cross-frequency dependence overlap, without interpreting rank agreement as an absence of additional information or as evidence of identical community partitions. This comparison concerns dependence representations across frequency bands, rather than the directed information transfer between market communities studied using transfer-entropy methods [29]. Second, it adapts a WaveClust-style framework to financial returns using a dual-power affinity construction that processes positive and negative similarities separately before nonnegative spectral clustering. The reported configuration attains ARI =0.381 relative to Shenwan sectors—comparable to the strongest correlation-based baseline—and yields 25 communities with no singletons. This outcome characterizes the reported configuration rather than a general guarantee of the method. Cross-band rank agreement and the observed sensitivity to layer-distance weighting are consistent with a shared dependence backbone, while denoising and common-factor explanations remain interpretations rather than mechanisms established by the analysis.

The remainder of the paper is organized as follows. Section 2 describes the data, preprocessing, MODWT decomposition, cross-scale nonnegative affinity construction, spectral community detection, and the evaluation protocol. Section 3 presents the grid-search setup, scale-dependent industry alignment across the sample windows, cross-scale weighting and dependence-overlap analyses, a grid-wide robustness check, the baseline comparison, and an information-based dependence measure evaluated under market and industry factor controls. Section 4 reports network and community characteristics. Section 5 discusses cross-frequency dependence overlap, the denoising and common-factor interpretations, and their limitations. Section 6 summarizes the findings.

## 2. Method

### 2.1. Data and Preprocessing Pipeline

The analysis uses a daily closing-price panel for Chinese A-share stocks sourced from Tushare Pro [30]. After alignment on observed trading dates, the raw price matrix contains 5466 stocks. The analysis applies four nested time windows that share a common 2025 endpoint and extend progressively further back—2019–2025, 2021–2025, 2023–2025, and 2024–2025—to examine sensitivity to the observation window and the associated eligible stock universe. A quality filter retains only stocks with at least 95% price coverage and a flat-return rate below 5%, requiring a minimum of 1000 eligible stocks per window. Because the coverage filter is easier to pass over shorter spans, shorter windows admit more eligible stocks; after filtering, the longest window (2019–2025) retains 2773 stocks across 1699 trading days, while the shortest (2024–2025) retains 4612. Changes across windows therefore reflect both the length of the observation period and the composition of the eligible universe.

The preprocessing pipeline consists of five steps.

Step 1 computes log returns.

(1) $$r_{t}=\ln\left(P_{t}\right)-\ln\left(P_{t-1}\right)$$

Step 2 applies two-sided winsorization at a quantile level α to limit the influence of extreme returns.

(2) $${\tilde{r}}_{i,t}=\left\{\begin{array}{cc} q_{\alpha }^{\left(i\right)} & \mathrm{if}\;r_{i,t}<q_{\alpha }^{\left(i\right)}\\ r_{i,t} & \mathrm{if}\;q_{\alpha }^{\left(i\right)}\leq r_{i,t}\leq q_{1-\alpha }^{\left(i\right)}\\ q_{1-\alpha }^{\left(i\right)} & \mathrm{if}\;r_{i,t}>q_{1-\alpha }^{\left(i\right)} \end{array}\right.$$

Step 3 applies stock-wise z-score standardization.

(3) $$z_{i,t}=\frac{{\tilde{r}}_{i,t}-\mu_{i}}{\sigma_{i}+\epsilon }$$

where $\mu_{i}$ and $\sigma_{i}$ are the mean and standard deviation of the winsorized series and ϵ is a small stabilizing constant.

Step 4 removes near-constant series and replaces residual infinite or missing values with zero.

Step 5 applies the MODWT directly to the standardized return series.

### 2.2. Shift-Invariant Decomposition via MODWT

We use the maximal overlap discrete wavelet transform (MODWT) [16], a shift-invariant variant of the multiresolution analysis of Mallat [15], to separate long-run trends from short-run fluctuations. In the decimated DWT, the cascade of low-pass and high-pass filters $\left\{h_{l}\right\}$ and $\left\{g_{l}\right\}$ followed by dyadic downsampling recursively produces approximation coefficients $cA_{j}$ and detail coefficients $cD_{j}$:

(4) $$\begin{array}{cc} cA_{j+1}\left[p\right] & =\sum_{l}h\left[l-2p\right]\cdot cA_{j}\left[l\right] \end{array}$$

(5) $$\begin{array}{cc} cD_{j+1}\left[p\right] & =\sum_{l}g\left[l-2p\right]\cdot cA_{j}\left[l\right] \end{array}$$

with $cA_{0}=z_{i,t}$. The decimation operator halves the sequence length at every level, which destroys time alignment across scales and makes the transform sensitive to circular shifts of the input.

The MODWT removes the downsampling step and rescales the filters by $\sqrt{2}$ at each level. Letting ${\tilde{h}}_{l}=h_{l}/\sqrt{2}$ and ${\tilde{g}}_{l}=g_{l}/\sqrt{2}$ denote the MODWT scaling and wavelet filters, the cascade becomes

(6) $$\begin{array}{cc} {\tilde{V}}_{j+1,t} & =\sum_{l=0}^{L-1}{\tilde{h}}_{l}\;{\tilde{V}}_{j,\;t-2^{j}l} \end{array}$$

(7) $$\begin{array}{cc} {\tilde{W}}_{j+1,t} & =\sum_{l=0}^{L-1}{\tilde{g}}_{l}\;{\tilde{V}}_{j,\;t-2^{j}l} \end{array}$$

where ${\tilde{V}}_{0,t}=z_{i,t}$ is the standardized return series, *L* is the filter length, *N* is the sample size, and $2^{j}$ in the index controls the dilated stride at level *j* (the à trous, or hole-insertion, scheme). Indices that fall outside [1,N] are supplied by symmetric reflection of the series at its boundaries, avoiding an assumption of periodic continuation. Boundary-affected coefficients are retained in similarity construction, and the extent of boundary influence depends on the wavelet filter and decomposition level. The level-*J* outputs are the approximation $cA_{J}={\left\{{\tilde{V}}_{J,t}\right\}}_{t=1}^{N}$ and the detail $cD_{j}={\left\{{\tilde{W}}_{j,t}\right\}}_{t=1}^{N}$ for j=1,…,J. Because no decimation is applied, every coefficient series retains the original length *N*, so coefficients at different scales remain time-aligned.

The MODWT is shift-invariant in the sense that a shift of the input produces the same shift in every output band, and it preserves an additive multiresolution analysis: $z_{i,t}={\tilde{V}}_{J,t}+\sum_{j=1}^{J}{\tilde{W}}_{j,t}$ when reconstructed with the corresponding inverse synthesis filters. The implementation uses PyWavelets [31] (version ≥ 1.4) with symmetric boundary reflection.

At decomposition level *J*, the MODWT yields J+1 frequency subbands: $cA_{J}$ captures fluctuations at periods $\geq 2^{J+1}$ trading days, while $cD_{j}$ isolates the band with periods in $\left[2^{j},2^{j+1}\right]$ trading days. For example, J=2 produces three subbands: $cA_{2}$ (low-frequency trend, ≥8 days), $cD_{2}$ (mid-frequency, 4–8 days), and $cD_{1}$ (high-frequency, 2–4 days). The decomposition level is the central experimental axis of this study: it is varied across J=2,…,6 to examine how industry alignment changes under the specified decomposition and fusion procedure. Changing *J* changes both the approximation band and the set of detail bands entering the fusion operator, so the resulting ARI pattern cannot by itself isolate a property of the underlying frequency bands.

In this study, four wavelet bases are tested: Daubechies-4 (Db4), Symlet-2 (Sym2), Symlet-4 (Sym4), and Coiflet-1 (Coif1). All four are compactly supported orthogonal filters and provide a perfect-reconstruction multiresolution analysis; they differ in vanishing moments and time–frequency localization, so comparing them assesses the sensitivity of the observed industry-alignment pattern to the choice of wavelet filter. After decomposition, each coefficient matrix is L2-normalized row-wise so that cosine similarity measures angular proximity in wavelet-coefficient space.

### 2.3. Cross-Scale Dual-Channel Network Construction and Spectral Community Detection

The network construction follows the WaveClust principle [22] that genuine associations should persist across multiple frequency bands, extended here with a dual-channel backend that treats positive and negative co-movement in separate channels before fusion. For stocks *i* and *j*, the cosine similarity at each frequency band *l* is

(8) $$C_{ij}^{\left(l\right)}=\frac{w_{i}^{\left(l\right)}\cdot w_{j}^{\left(l\right)}}{∥w_{i}^{\left(l\right)}∥\;∥w_{j}^{\left(l\right)}∥}$$

where $w^{\left(l\right)}$ is the L2-normalized wavelet-coefficient vector of a stock at band *l*. Each similarity is split into a positive and a negative part, $s_{ij}^{+,(l)}=\max\left(C_{ij}^{\left(l\right)},0\right)$ and $s_{ij}^{-,(l)}=\max\left(-C_{ij}^{\left(l\right)},0\right)$, so that co-moving and anti-correlated pairs contribute through separate channels.

The cross-scale dual-channel score fuses these channels across frequency bands. For a *J*-level decomposition, the low-frequency approximation $cA_{J}$ is paired with each detail subband $cD_{l}$ (l=1,…,J), and the score keeps the strongest cross-band association:

(9) $$\begin{array}{cc} S_{ij}^{\mathrm{dual}} & =\max_{l=1,\ldots ,J}\{\left(k\cdot l+1\right)[\sqrt{s_{ij}^{+,(cA_{J})}\cdot s_{ij}^{+,(cD_{l})}}\\ \; & \;+\;w_{\mathrm{neg}}\cdot \sqrt{s_{ij}^{-,(cA_{J})}\cdot s_{ij}^{-,(cD_{l})}}]\} \end{array}$$

where $w_{\mathrm{neg}}$ scales the contribution of anti-correlated pairs and k≥0 is the layer-distance weight: when k=0 all cross-scale pairs contribute equally, while k>0 gives greater weight to associations that persist into more distant detail bands. The score matrix is symmetrized via $S^{\mathrm{dual}}\leftarrow \max\left(S^{\mathrm{dual}},{\left(S^{\mathrm{dual}}\right)}^{⊤}\right)$ with the diagonal set to zero. Signed-graph methods that model negative edges explicitly have been shown to improve community recovery in correlation-based networks [32], where anti-correlated assets carry structural information unavailable to positive-only affinity matrices. The construction used here is related but weaker, and the distinction matters. Before fusion, the two channels are separate, so whether a band-level dependence is positive or negative influences the score through $s^{+}$ and $s^{-}$ individually. After fusion, both channels contribute additively to a single nonnegative value, and Equation (9) satisfies $S_{ij}^{\mathrm{dual}}\geq 0$ for all pairs. The edge sign is therefore not recoverable from the fused score: $S_{ij}^{\mathrm{dual}}$ alone does not determine whether the underlying dependence was positive or negative. The matrix passed to spectral clustering is a nonnegative affinity, not a signed graph, and we do not claim the properties of signed-graph spectral methods for it.

The fused score is mapped to a nonnegative affinity matrix and then transformed by a power map,

(10) $$A_{ij}=\frac{\max(S_{ij}^{\mathrm{dual}},0)}{\max_{u,v}S_{uv}^{\mathrm{dual}}}\;A_{ij}^{\left(\gamma \right)}=A_{ij}^{\gamma }$$

with the diagonal reset to one. The normalization places every off-diagonal entry in [0,1], and the tested values γ∈{0.35,0.40} satisfy 0<γ<1. For $0<A_{ij}<1$ this gives $A_{ij}^{\gamma }>A_{ij}$, with weak affinities raised proportionally more than strong ones, so the transform *compresses* the contrast between strong and weak associations rather than sharpening it. Its role in this pipeline is to keep moderately weighted edges from being dominated by the strongest ones in the normalized Laplacian; we treat γ as a hyperparameter swept over the grid and claim no further property for the map. Communities are then recovered by normalized spectral clustering [33]: we form the symmetric normalized affinity $L_{\mathrm{sym}}=D^{-1/2}A^{\left(\gamma \right)}D^{-1/2}$, embed stocks in the space of its leading eigenvectors, renormalize each embedding to unit length, and assign labels with the discretize algorithm under a fixed random seed. The number of communities *K* is not inferred internally but is treated as a hyperparameter swept over a range bracketing the 31 Shenwan first-level industries.

The method therefore exposes four free hyperparameters: the layer-distance weight *k* (how strongly associations in more distant detail bands are upweighted), the negative weight $w_{\mathrm{neg}}$ (the contribution of anti-correlated pairs), the power parameter γ (the contrast compression), and the community count *K*. These, together with the wavelet basis and decomposition level, define the configuration space and tested ranges reported in the experimental setup.

*Remark.* The dual-channel backend, used together with the price-quality filter, is designed to reduce singleton-collapse risk relative to naïve full-sample positive-only spectral approaches; in the reported reference configuration it yields a balanced partition with no singleton communities.

### 2.4. Clustering Evaluation Against Shenwan Taxonomy

Clustering quality is evaluated against Shenwan industry classifications at three granularity levels (SW1: 31 industries, SW2: 129 industries, SW3: 318 industries). The ARI [34] is computed only on stocks with valid Shenwan labels.

Let $Y=\{Y_{1},\ldots ,Y_{R}\}$ denote the industry partition (Shenwan labels) and $C=\{C_{1},\ldots ,C_{K}\}$ the clustering partition over *n* stocks. Define the contingency matrix entries $n_{ij}=\left|Y_{i}\cap C_{j}\right|$, the row sums $a_{i}=\sum_{j}n_{ij}$, and the column sums $b_{j}=\sum_{i}n_{ij}$. The ARI is

(11) ARI=∑ijnij2−∑iai2∑jbj2/n212∑iai2+∑jbj2−∑iai2∑jbj2/n2

ARI takes values in [−1,1]: zero corresponds to chance agreement and one to a perfect match with the industry partition. Unlike the unadjusted Rand index, ARI is corrected for the agreement expected by chance under a hypergeometric null on the contingency table, which makes it directly comparable across partitions with different cluster counts.

ARI is used as an external validation measure. Shenwan labels are introduced after the clustering partition has been determined, and an algorithmic constraint enforces this separation: no label enters the preprocessing, the wavelet decomposition, the affinity construction, or the assignment step of any individual partition. The labels are not, however, confined to post-hoc validation. They inform the range of candidate community counts *K*, which brackets the 31 first-level industries, and the reported reference configuration is selected by comparing ARI across the grid. ARI therefore also functions as a model-selection criterion at the level of configurations, and the reported reference value characterizes a configuration chosen with reference to the labels rather than an out-of-sample outcome.

Sensitivity to the observation period is examined across four nested windows of increasing length (Table 1), each subject to the same price-quality filter. Because the windows are nested and the eligible universe changes with window length, this design does not isolate the effect of the sample period from that of universe composition; it examines whether the scale-dependent pattern persists as both vary together.

## 3. Experimental Setup and Results

### 3.1. Experimental Setup

We conduct a systematic grid search over the wavelet basis, decomposition level, and the four clustering hyperparameters of the dual-channel spectral backend (*k*, $w_{\mathrm{neg}}$, γ, and the community count *K*), evaluated across the four time windows. The grid uses wavelet bases {Db4, Sym2, Sym4, Coif1}, decomposition levels J∈{2,3,4,5,6}, layer-distance weights k∈{0,0.5,1.0,1.5}, winsorization levels {3%, 3.5%}, power parameters γ∈{0.35,0.40}, negative weights $w_{\mathrm{neg}}\in \left\{0.75,1.0,1.25\right\}$, and community counts K∈{25,31,33,35}. In total this yields 15,360 configurations (3840 per window). All trials enforce strict separation between the construction of an individual partition and label evaluation: Shenwan labels enter after a partition has been fixed, and are used to compare configurations across the grid.

The price-quality filter requires ≥95% coverage and ≤5% flat-return rate, retaining only actively traded stocks. The four windows and their eligible stock counts are listed in Table 1.

### 3.2. Overview: Central Results and Robustness Exercises

The empirical message of this section is fourfold. First, industry alignment declines as the decomposition becomes deeper. Second, dependence rankings nevertheless remain substantially related across bands. Third, independently clustered bands are far from identical. Fourth, common market and industry components explain part, but not all, of the cross-frequency overlap. The following subsection establishes the first point, and Section 3.4 and Section 3.7 the second through fourth. The remaining subsections are supporting exercises rather than new findings: Section 3.5 checks that the first point holds across the configuration ensemble, and Section 3.6 places the absolute ARI level in context against correlation-based alternatives.

### 3.3. Scale-Dependent Community Structure

The central empirical finding is a monotonic decay of industry alignment with decomposition level. This decay is an empirical property of the particular pipeline studied here—MODWT decomposition, weighted-maximum dual-channel fusion, and normalized spectral clustering—and is not interpreted as a general consequence of increasing frequency resolution. Table 2 reports the best ARI achieved at each level in the longest window (2019–2025, 2773 stocks), together with the resulting community count and largest community size.

ARI decays from 0.381 at level 2 to 0.139 at level 6, a 64% reduction. This is a statement about agreement with the Shenwan first-level taxonomy alone: shallower decompositions, which fuse a more compact set of frequency bands, yield the partitions most closely aligned with industry labels. Communities recovered at deeper levels may still carry structure organized along dimensions other than industry, and the present design cannot exclude that; the decline is therefore not used to infer that the deeper bands are uninformative.

**Figure 1:**
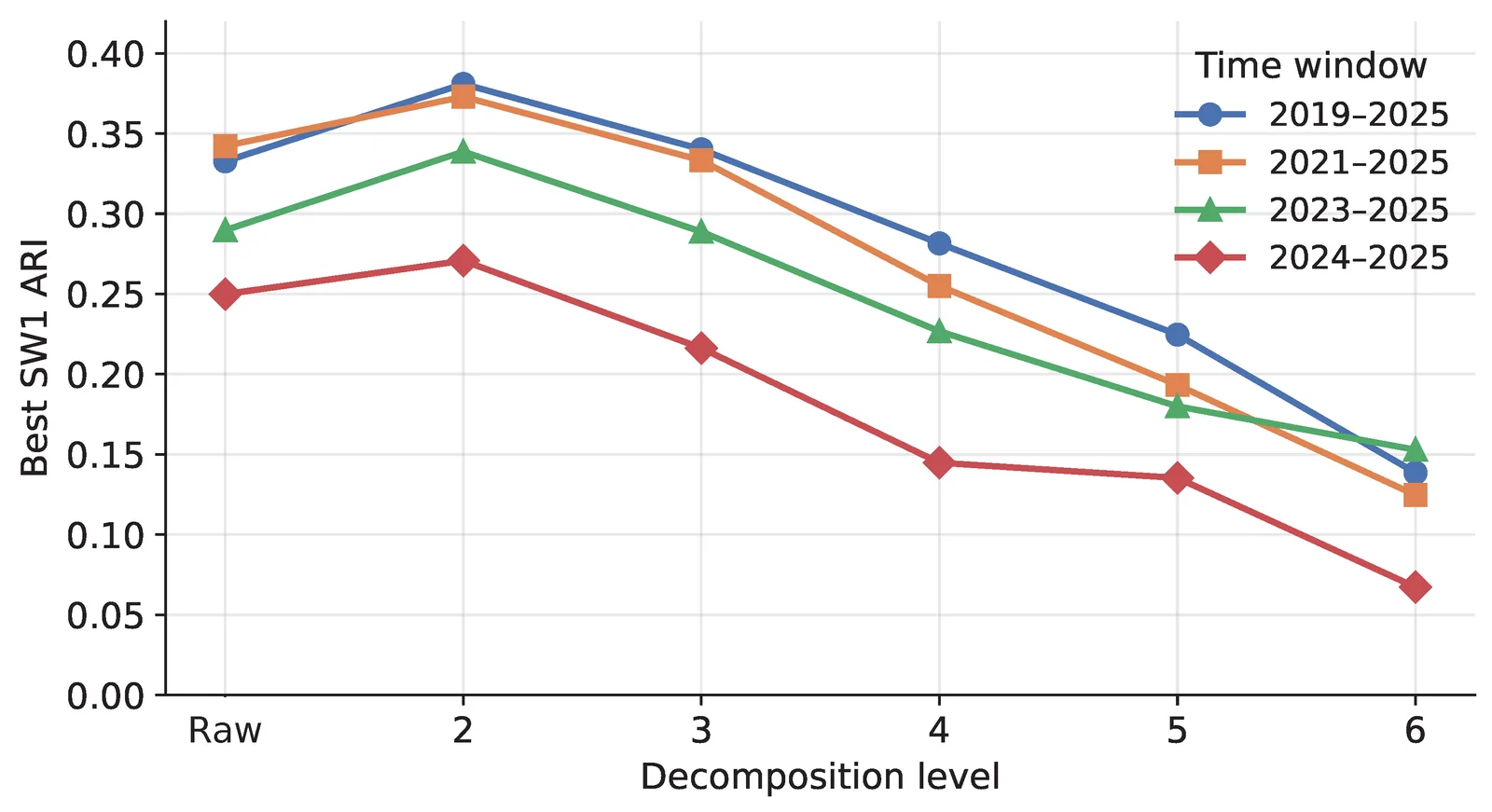
Scale-dependent community structure: best ARI peaks at decomposition level 2 and then decays monotonically with increasing level across all four time windows. The raw point corresponds to the no-wavelet baseline (clustering run directly on the raw return series); in every window a shallow wavelet decomposition (level 2) outperforms it, after which the monotonic decay is preserved regardless of window length or eligible stock count.

Figure 1 compares the level-wise pattern across all four observation windows. In every window the best ARI peaks at level 2 and declines monotonically thereafter, preserving the ordering J=2>J=3>J=4>J=5>J=6, while the level-2 optimum ranges from 0.381 (2019–2025; Sym2, k=1.5) to 0.271 (2024–2025; Sym2, k=0.5). In both of these windows the optimum is attained by the same wavelet basis but at a different layer-distance weight—consistent with the weak sensitivity to *k*: when the ARI response to *k* is nearly flat, the location of the best-performing *k* need not be stable across samples. We use scale near-invariance as a descriptive shorthand for this preserved ordering: the level ordering of industry alignment is stable across the tested sample periods and model settings. This cross-window stability of the level ordering provides the temporal-robustness check of the scale-dependent pattern.

As a supplementary baseline, we also evaluate the pipeline without any wavelet decomposition (raw, clustering run directly on the raw return series). The best ARI in this no-wavelet setting is 0.333 (2019–2025), 0.342 (2021–2025), 0.290 (2023–2025), and 0.250 (2024–2025)—below the level-2 value in every window (Figure 1). A shallow MODWT decomposition therefore provides a consistent gain over the raw-return baseline in all four windows, consistent with the multiresolution step contributing structural signal beyond what the unfiltered series carries; the gain peaks at level 2 and is eroded by deeper decompositions. The margin is modest—0.381 against 0.333 in 2019–2025—and both are best-over-grid values, so the ordering is more informative than its size.

The ordering is robust, but absolute ARI levels are not directly comparable across windows: observation length and universe composition change together across the nested windows (Table 1), so cross-window differences in absolute ARI reflect both effects and cannot be attributed to window length alone. A supplementary check on three non-overlapping periods with a fixed stock universe reproduces the shallow-to-deep ordering, though in one period the decline is not monotonic at every intermediate level.

Since level 2 attains the best ARI in every window, we adopt the best level-2 configuration in the longest window (Sym2, k=1.5; 2019–2025) as the reference configuration for the subsequent network analysis.

### 3.4. Cross-Scale Weighting and Cross-Frequency Dependence Overlap

**Figure 2:**
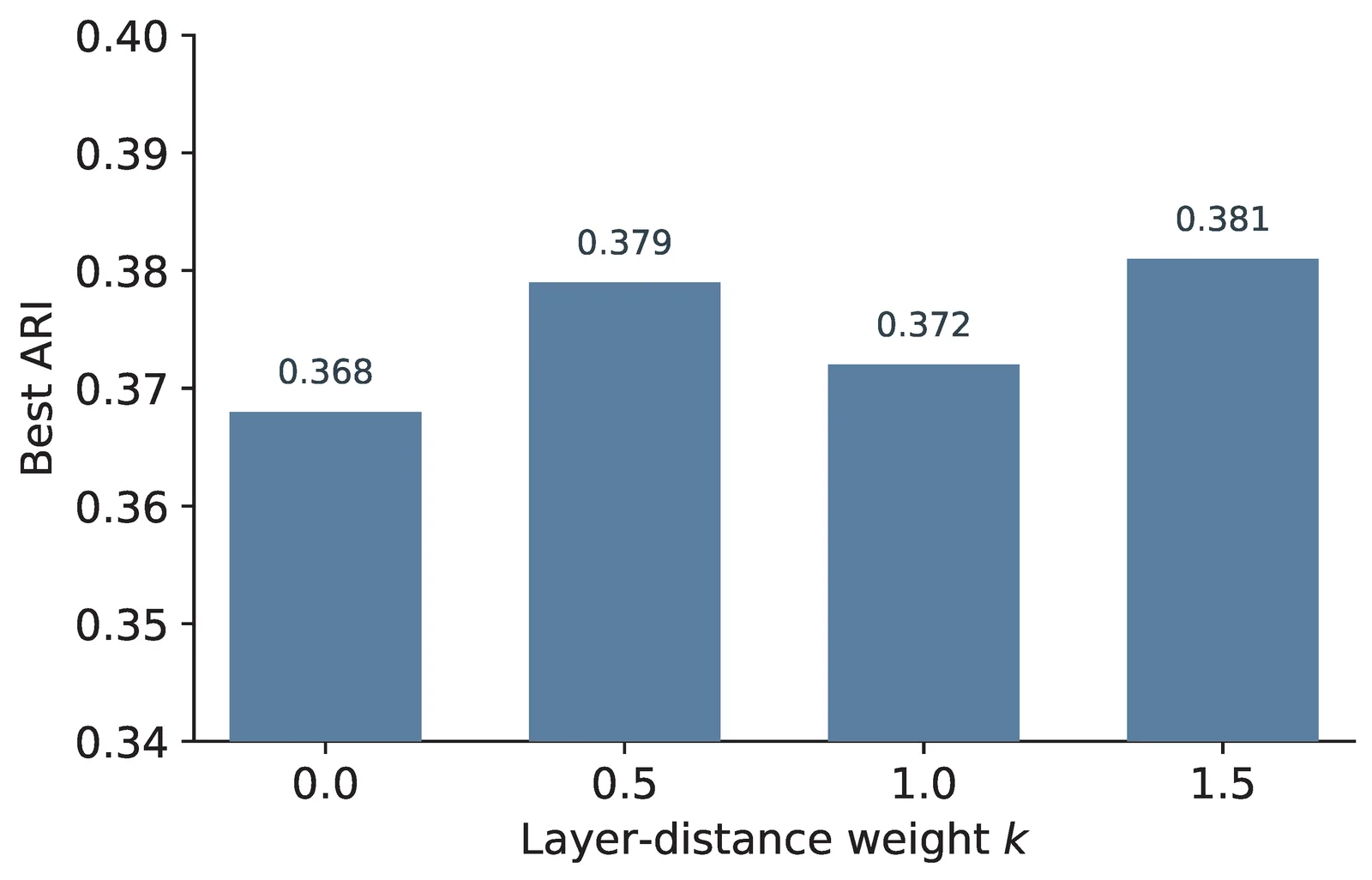
Limited sensitivity of best ARI to the layer-distance weight *k* at level 2 (2019–2025): best ARI across all configurations at each *k*, shown on a 0.34–0.40 axis. The narrow spread (max − min =0.013, 3.4%) is consistent with substantial cross-frequency dependence overlap.

A central question for any multiresolution framework is whether weighting associations by their frequency-band distance improves community recovery. Figure 2 shows that, at level 2 in the 2019–2025 window, the layer-distance weight *k* has almost no effect: best ARI varies by only 3.4% across the full range of *k*. Equal-weight fusion (k=0) also attains the highest *mean* ARI across the grid, so the additional complexity of distance weighting yields no robust gain over the simplest fusion rule. This near-invariance to *k* indicates that the bands rank stock pairs similarly enough that reweighting them by band distance barely moves the fused score. How much information they share is a separate question, addressed in Section 3.7.

**Figure 3:**
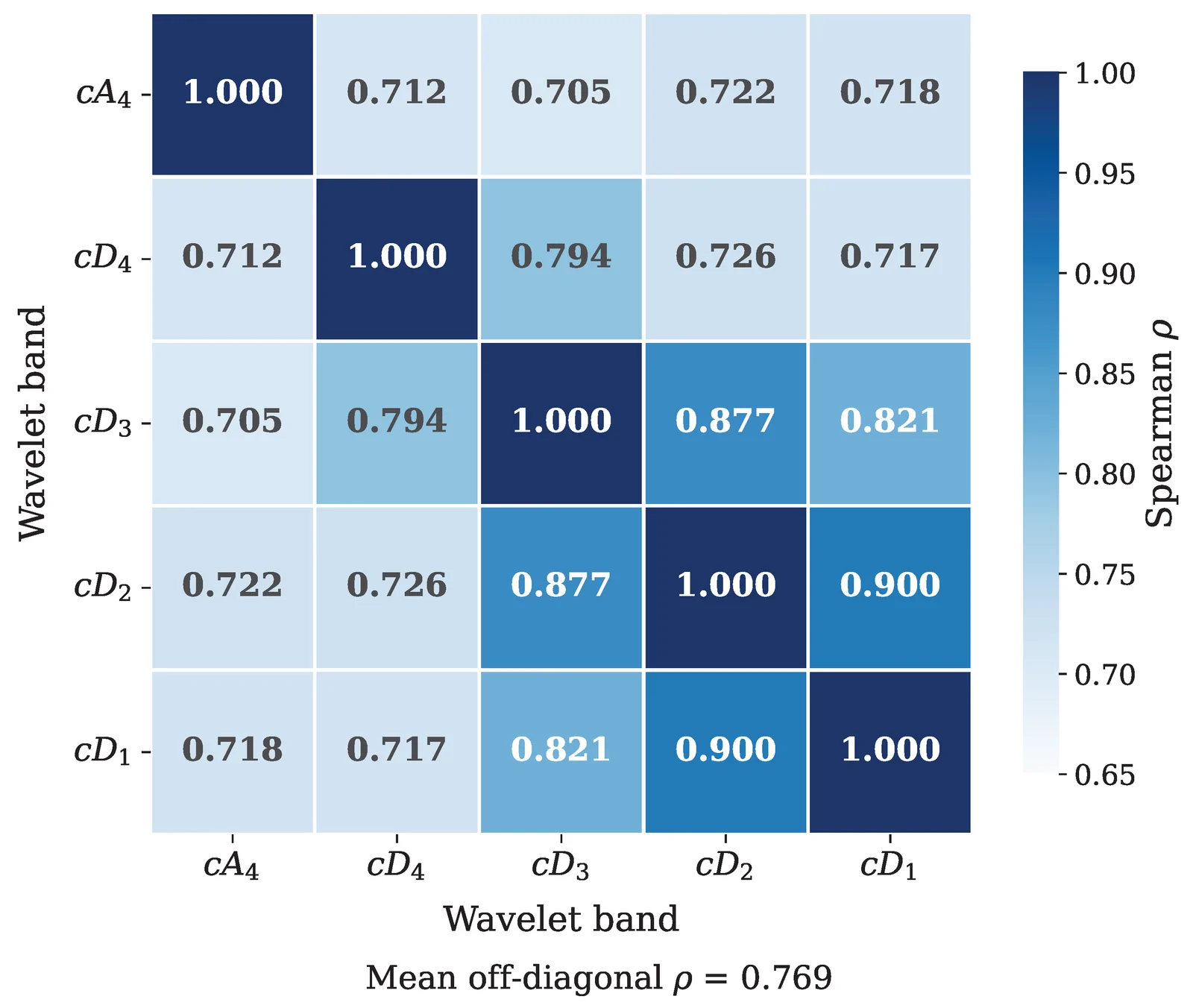
Cross-frequency Spearman rank correlation between MODWT band-wise pair-similarity vectors on the 2019–2025 universe. Off-diagonal entries quantify how consistently two frequency bands order the same stock pairs; the mean off-diagonal value is 0.769, quantifying rank agreement between band-wise similarity vectors; agreement with the industry taxonomy is a separate quantity, reported in Table 2.

Direct evidence of cross-frequency rank agreement comes from measuring how consistently different bands order the same stock pairs. Figure 3 reports the Spearman rank correlation between the upper-triangular similarity vectors at each MODWT band ($cA_{4}$, $cD_{4}$, $cD_{3}$, $cD_{2}$, $cD_{1}$) on the price-quality-filtered universe. The mean off-diagonal Spearman ρ is 0.769, with the smallest entry at $\rho (cA_{4},cD_{3})=0.705$ and the largest at $\rho (cD_{1},cD_{2})=0.900$. Adjacent bands share more structure than distant ones, yet even the weakest pair exceeds ρ=0.70, leaving the layer-distance weight little room to reshape the fused score—so the level-2 ARI plateau in Figure 2 is consistent with substantial dependence overlap across frequency bands rather than with an artifact of the fusion rule. Rank agreement of this magnitude establishes that the bands order stock pairs similarly; it does not establish that the additional bands carry no further information.

### 3.5. Grid-Wide Robustness

**Figure 4:**
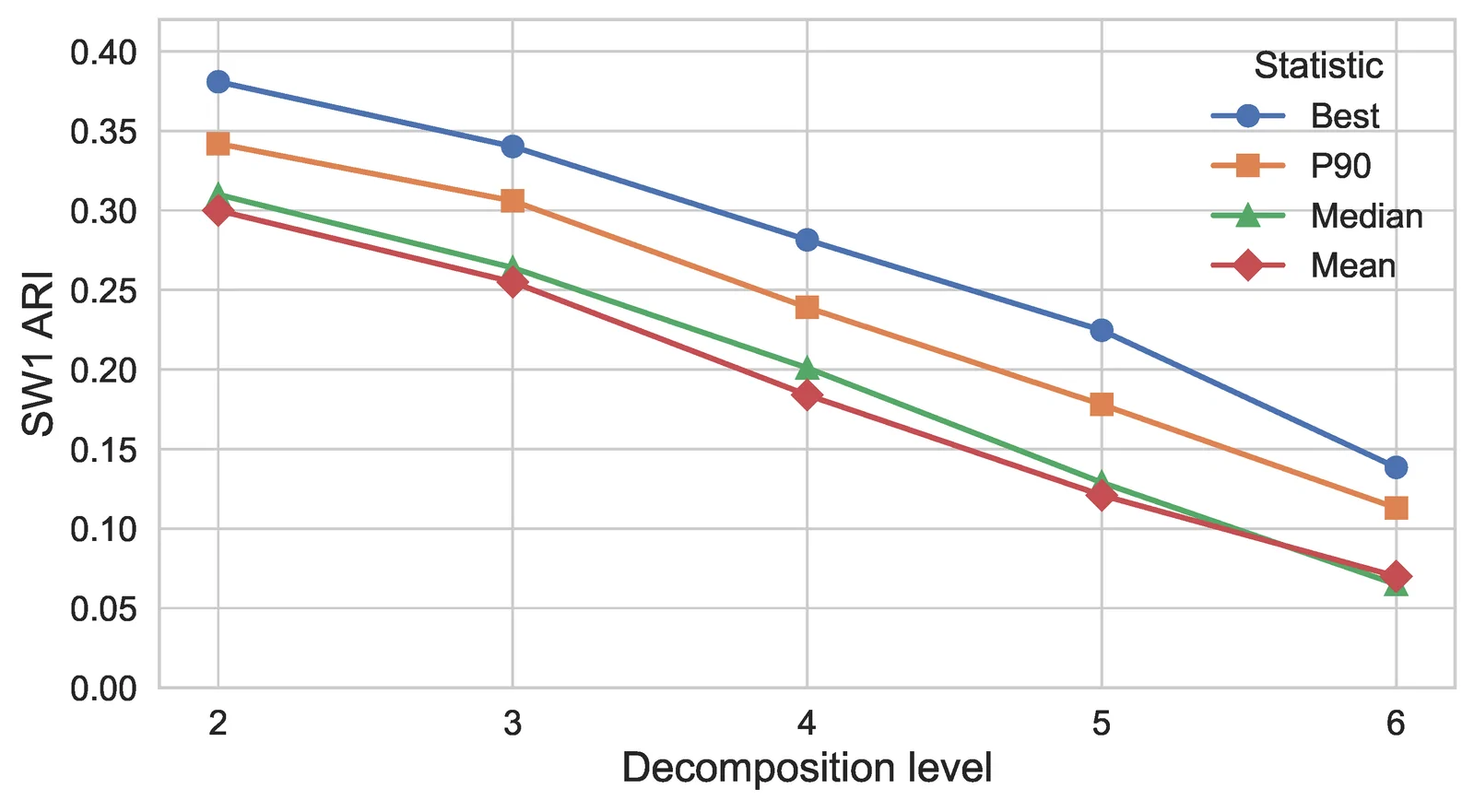
Distribution of the adjusted Rand index (ARI) against the Shenwan first-level taxonomy as a function of MODWT decomposition level, pooled over the four time windows (3072 configurations per level). For each level we report four summary statistics of this configuration ensemble—the grid-wide mean, the median, the 90th percentile (P90), and the single best (maximum) ARI. All four statistics decrease monotonically from level 2 to level 6 (e.g., mean ARI 0.300 → 0.070; P90 0.342 → 0.113), confirming that the level decay is a property of the full configuration distribution rather than an artifact of selecting a single favorable hyperparameter setting.

So far, the evidence for the level decay rests on the best configuration at each level. To determine whether the observed level dependence is restricted to a small set of favorable hyperparameter settings, we examine the full ensemble of 15,360 trials (3072 per level, pooled over the four windows). Across this ensemble, the mean, median, 90th percentile, and maximum ARI all decrease monotonically from level 2 to level 6 (mean 0.300 → 0.070; P90 0.342 → 0.113; Figure 4). The scale-dependent decline is therefore not tied to any particular hyperparameter combination but is a grid-wide property of the experiment.

### 3.6. Comparison with Baselines

We compare against four fundamentally different clustering approaches on the same 2019–2025 universe (2773 stocks after price-quality filtering): Pearson correlation with normalized spectral clustering, hierarchical clustering on correlation distance, *k*-means on raw return vectors, and *k*-means on PCA-reduced returns (50 components). All methods use identical preprocessing (log returns, winsorization, z-score) and identical post hoc evaluation against Shenwan SW1 labels using ARI. The dual-channel spectral backend’s main role is not to maximize a single metric but to provide a stable unified framework for the multiresolution diagnostic; the baseline comparison contextualizes its absolute performance.

**Figure 5:**
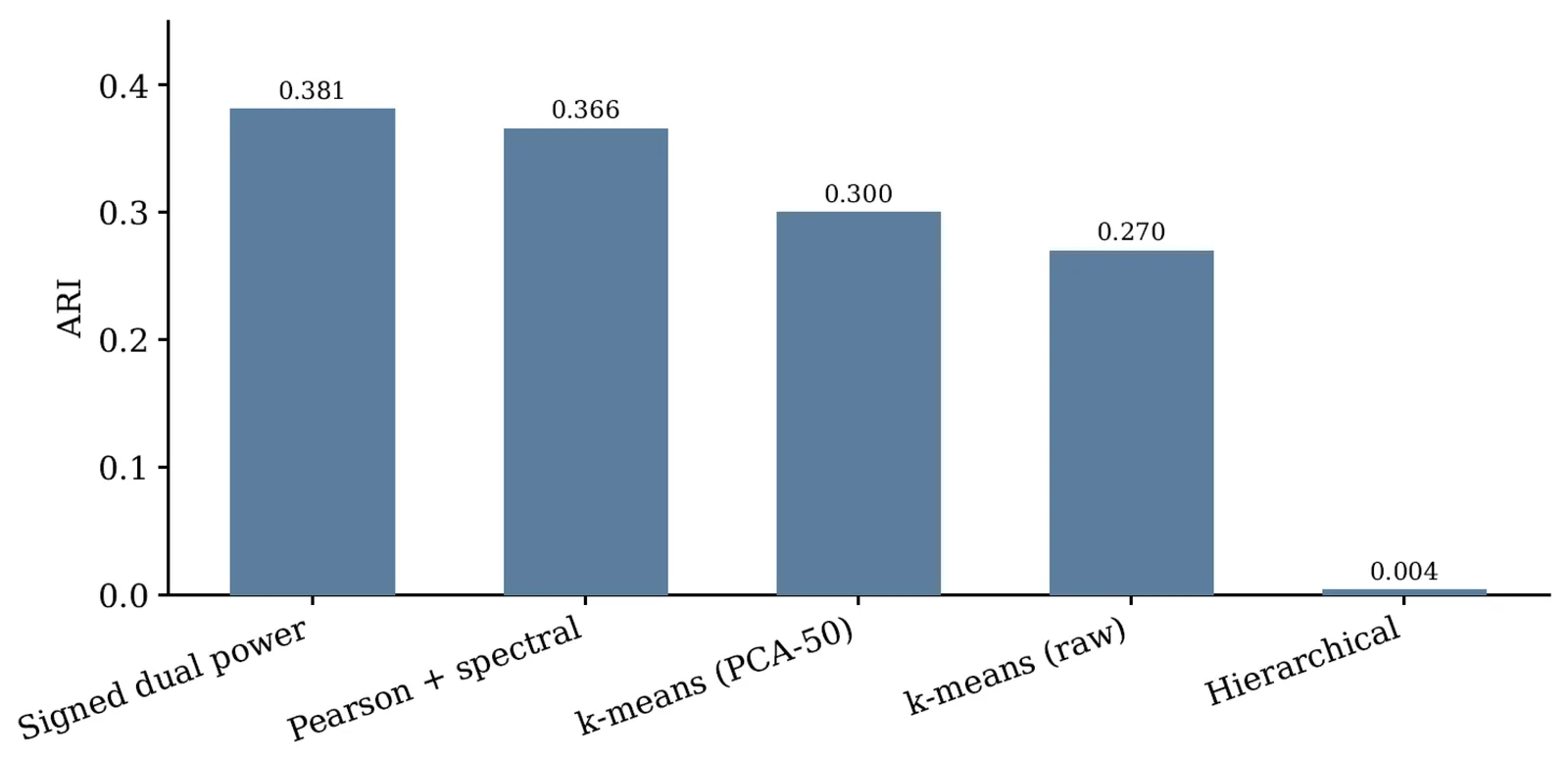
Comparison of ARI across the proposed method and four baseline clustering approaches on the 2019–2025 stock universe.

As shown in Figure 5, the proposed dual-channel spectral method attains an ARI of 0.381, comparable to Pearson correlation with spectral clustering (0.366). The non-wavelet baselines also achieve substantial ARI, indicating that the broad community structure is detectable from raw correlations; the wavelet-based pipeline does not claim a large absolute performance gain, but it provides the multiresolution decomposition that enables the level-decay analysis.

Hierarchical clustering with average linkage on correlation distance collapses (ARI =0.004), reproducing the topological-degeneration pattern reported in earlier work: a few tightly connected pairs absorb most of the universe before the target cluster count is reached. Spectral and partitional methods avoid this failure mode.

### 3.7. Information-Based Dependence Measures and Factor Controls

The decomposition-level sweep measures agreement with an external taxonomy, and the Spearman diagnostic measures rank agreement between band-wise similarity vectors. Neither speaks to how much information two bands share, nor to how much of the shared structure is attributable to common exposures. This subsection addresses both with an information-based measure and with two factor controls.

*Information-based dependence measure.* For an ordered pair of bands we compare the two upper-triangular stock-pair similarity vectors, each of length n(n−1)/2. Both vectors are discretized into 64 equal-frequency bins: values are ranked with a stable sort and bin *b* receives the ranks in [bN/64,(b+1)N/64), so every bin holds ⌊N/64⌋ or ⌈N/64⌉ observations and ties are broken by the stable order. Writing *X* and *Y* for the resulting discrete variables, mutual information is

(12) $$I\left(X;Y\right)=\sum_{x}\sum_{y}p\left(x,y\right)\;\ln\;\frac{p(x,y)}{p(x)\;p(y)}$$

in nats, and normalized mutual information uses the arithmetic mean of the two marginal entropies,

(13) $$\mathrm{NMI}\left(X;Y\right)=\frac{2\;I(X;Y)}{H(X)+H(Y)},\;H\left(X\right)=-\sum_{x}p\left(x\right)\ln p\left(x\right).$$

Equations (12) and (13) interact in a way that must be stated explicitly. Equal-frequency binning fixes both marginals at the uniform distribution over 64 bins, so H(X)=H(Y)=ln64 up to the rounding of *N* into bins, and Equation (13) reduces to NMI=I(X;Y)/ln64. Under this specification NMI is therefore a constant rescaling of MI rather than an independent measure, and we report the two as one quantity on two scales, not as two pieces of evidence. On the 2770-stock universe defined below, the mean off-diagonal MI across the five bands is 0.513 nats, giving a mean off-diagonal NMI of 0.123. Both figures are specific to the 64-bin equal-frequency discretization, so we use them comparatively across band pairs and factor-control branches rather than as absolute levels.

*Factor controls.* Three return branches enter the comparison. The *raw* branch is the series used throughout the paper. The *market-residual* branch replaces each series by the residual of a leave-one-out market regression,

(14) $$r_{i,t}=\alpha_{i}+\beta_{i}\;r_{m,t}^{\left(-i\right)}+\varepsilon_{i,t},\;r_{m,t}^{\left(-i\right)}=\frac{1}{N-1}\sum_{j\neq i}r_{j,t},$$

where, in Equation (14), the market proxy for stock *i* is the equal-weighted cross-sectional mean of all *other* stocks, so that the regressand is excluded from its own regressor. The regression is estimated by ordinary least squares on winsorized log returns before standardization, over the full 2019–2025 window with one $\left(\alpha_{i},\beta_{i}\right)$ pair per stock; residuals are then z-scored, so this branch and the raw branch differ only by the projection. The *sequential* branch additionally removes within-industry means from the standardized residuals, ${\tilde{\varepsilon }}_{i,t}=\varepsilon_{i,t}-{\bar{\varepsilon }}_{g(i),t}$, where g(i) is the industry of stock *i* and the mean is taken across that industry on each day; for equal-weighted groups this is the ordinary least squares projection onto industry indicators. The order is market first, then industry, and is not interchangeable. After the projection the largest absolute within-industry daily mean is $1.2\times 10^{-7}$, at the single-precision epsilon of the stored coefficients, confirming that it is executed to numerical precision.

Two specification details matter for interpretation. First, the industry groups used for the projection are the 106 groups of the Tushare industry field, not the 31 Shenwan first-level sectors against which ARI is evaluated; the removed variable and the evaluation label are therefore distinct, and the projection does not remove the evaluation target by construction. Second, the projection requires at least two stocks per industry group, which excludes three of the 2773 stocks—two whose industry group is a singleton and one without an industry label—so all branches in this subsection share a common universe of 2770 stocks.

**Figure 6:**
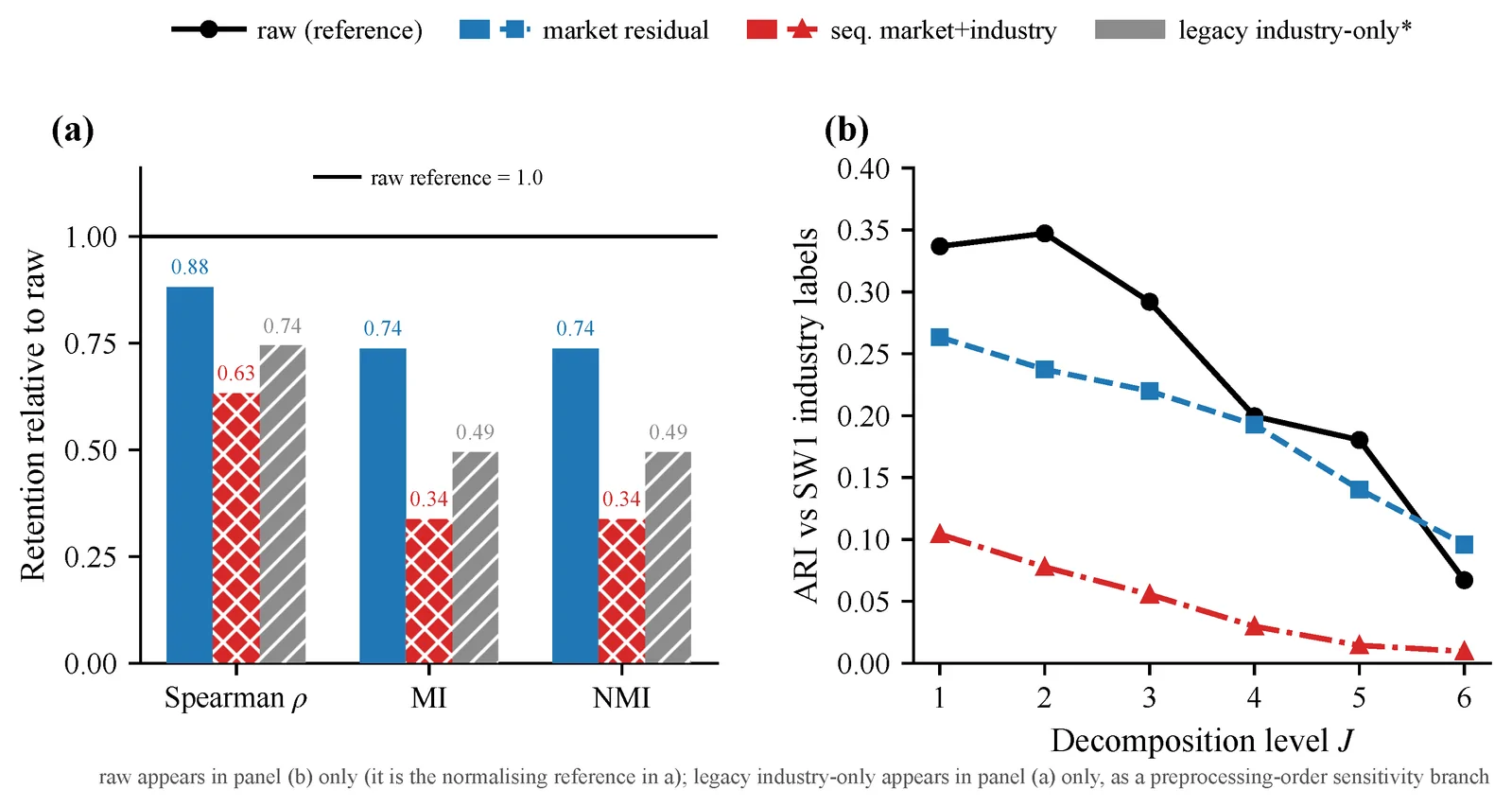
Cross-frequency dependence and level profile under factor controls, on a common universe of 2770 stocks. (**a**) Mean off-diagonal cross-band dependence per branch relative to the raw branch, on three measures (Spearman ρ, MI, NMI); the raw branch is the normalizing reference at 1.0 and is drawn as the horizontal line. MI and NMI retention coincide exactly because 64 equal-frequency bins fix both marginal entropies at ln64, making NMI a constant rescaling of MI (Equation (13)). Dependence panel: Coif1, J=4, winsorization 3.5%, 64 bins. The asterisk (*) identifies the legacy industry-only branch, which applies the industry projection before stock-wise standardization and is reported as a preprocessing-order sensitivity check. (**b**) ARI against Shenwan first-level labels by decomposition level for the three main branches. Clustering panel: Sym2, winsorization 3%, weighted-maximum fusion, k=1.5, $w_{\mathrm{neg}}=1.25$, γ=0.35, K=25, seed 42; one partition per branch–level cell and no repeats, so these values are single-configuration outcomes rather than the best-over-grid values of Table 2. The J=1 point is a one-level MODWT supplement with bands $cA_{1}$ and $cD_{1}$.

*Results.* Figure 6 reports both quantities. Mean cross-band Spearman correlation decreases from 0.769 for raw returns to 0.677 after market removal and 0.486 after sequential market-and-industry removal, retaining 88% and 63% of the raw value. The information-based measure falls proportionally further, retaining 74% and 34%; because NMI is a rescaling of MI here, the two retention values coincide by construction. Common exposures therefore account for part of the measured cross-band dependence, and more of the information-based dependence than of the rank agreement, while a substantial share of the rank agreement survives both projections. These are changes in two summaries of the similarity matrices; they quantify sensitivity to the two projections and do not identify a factor model or establish a single common-factor mechanism for the pattern.

The level profile in Figure 6b is measured under one fixed configuration rather than as a best-over-grid value, and the ARI levels are accordingly below those of Table 2. Within that fixed configuration, all four adjacent differences from J=2 to J=6 are negative in each of the three branches, so the decline in industry alignment with decomposition depth is not confined to raw returns and is not produced by the common market component alone. The market-residual branch lies below the raw branch at J=2 through J=5 but above it at J=6, so market removal does not lower industry alignment uniformly at every level. The sequential branch has low absolute ARI. Removing means within Tushare industry groups can attenuate return structure associated with Shenwan sectors to the extent that the two classifications overlap. Its ARI therefore describes the industry alignment remaining after this projection.

## 4. Network Structure and Community Characteristics

### 4.1. Reference Partition and Global Network Topology

The reference configuration (Sym2, level 2, k=1.5) in the 2019–2025 window assigns the 2773 eligible stocks to 25 communities. Post-hoc evaluation against Shenwan labels yields ARI =0.381 at the first level (31 industries). At finer Shenwan granularities, ARI decreases to 0.193 (SW2, 129 industries) and 0.116 (SW3, 318 industries), because a fixed count of 25 communities cannot resolve increasingly fine-grained industry distinctions.

This reference partition exceeds the level-2 grid-wide mean (0.300, pooled over the four windows) by 27% and the corresponding 90th percentile (0.342) by 11%. Part of this margin reflects the window itself rather than the hyperparameter choice, because the 2019–2025 window attains the highest absolute ARI among the four windows; even so, the high pooled mean indicates that the dual-channel dual-power method produces informative partitions across a wide range of settings.

The partition is also well balanced: no community is a singleton, the median community size is 115 stocks, and the largest community contains 220 stocks (7.9% of the universe). At the 99.6th-percentile reporting threshold, the corresponding graph contains 9704 edges (density 0.0025). This singleton-free balance contrasts sharply with a full-sample baseline (no price-quality filter, positive-only full-band similarity) that produced 345 singletons among 380 communities. Because the present pipeline changes several elements at once—price-quality filtering, the separate positive and negative similarity channels, the power transform, and the spectral assignment—this outcome cannot be attributed to any single component without a dedicated ablation. The absence of singletons means the spectral assignment does not degenerate, which makes the partition usable for the subsequent analysis; industry alignment is measured separately by ARI.

These statistics describe the network’s global organization but not how stocks from different industries are arranged within individual communities; we therefore turn to the most industry-diverse communities next.

### 4.2. Local Community Structure

**Figure 7:**
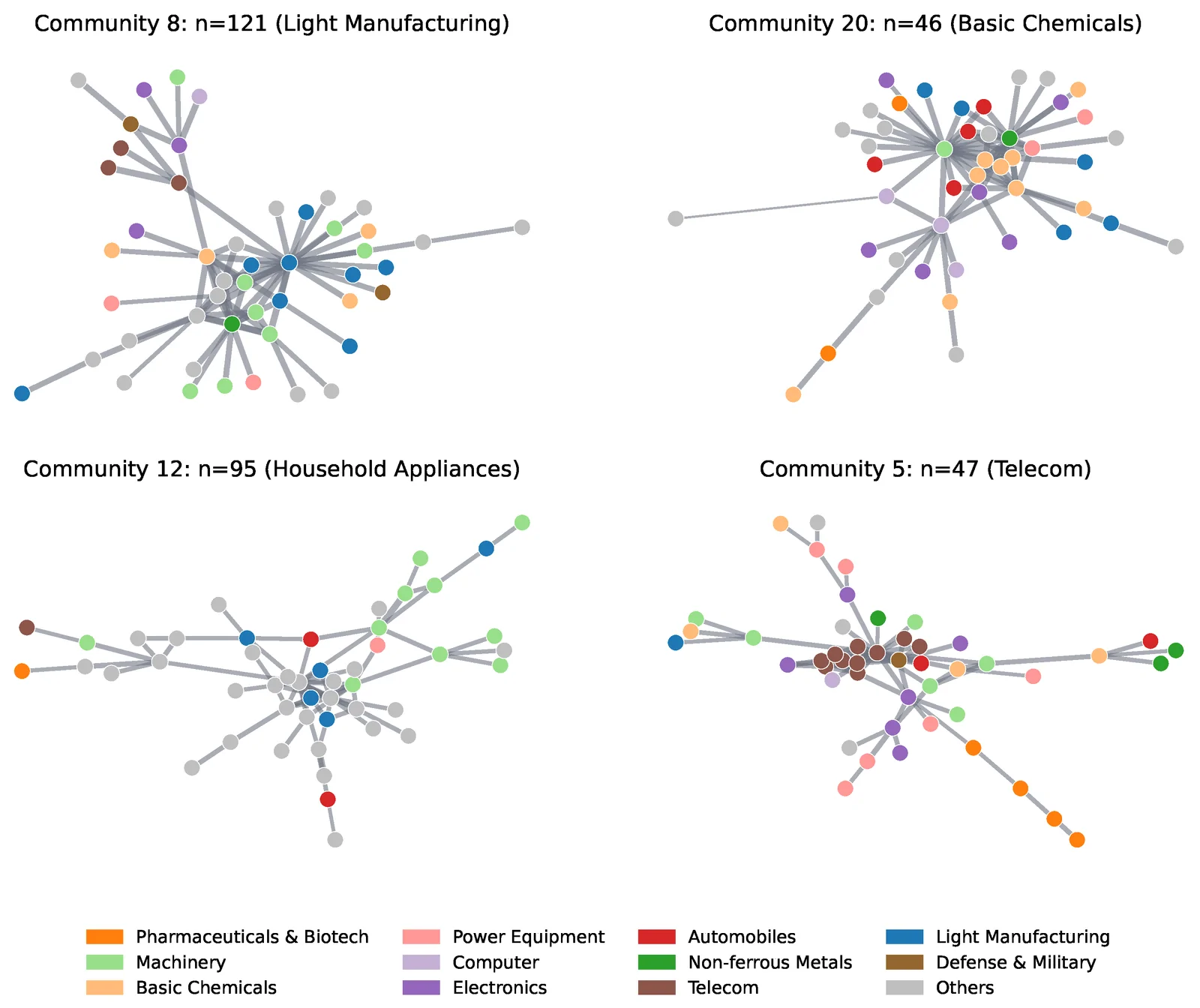
Local dependence structure of the four most industry-diverse communities in the reference partition (Sym2, level 2, k=1.5, 2019–2025), selected for spanning the largest number of Shenwan first-level industries. Nodes are stocks colored by Shenwan first-level industry; edges denote strong positive within-community return associations (maximum-spanning-tree backbone plus highest-correlation hub and residual links). Panel titles report the full community size *n* and its (weakly) dominant industry. Communities larger than 48 stocks are subsampled for readability. Each community mixes 14–25 industries with no sector exceeding 21% of members, yet retains a coherent strongly weighted core, suggesting that within-community co-movement is not reducible to single-industry membership.

Figure 7 visualizes the internal dependence structure of the four *most industry-diverse* communities in the reference partition—those whose membership spreads across the largest number of Shenwan first-level industries rather than concentrating in a single sector. Within each panel, nodes are individual stocks colored by their Shenwan first-level industry, and edges trace the strong positive within-community return associations recovered by a maximum-spanning-tree backbone augmented with the highest-correlation hub and residual links; node positions follow a force-directed layout so that tightly co-moving stocks are drawn together. To keep each panel legible, communities larger than 48 stocks are rendered from a fixed random subsample, while the reported size *n* refers to the full community.

These panels offer a stress test for the common-factor interpretation because they focus on communities that are least aligned with a single industry taxonomy. Each spans between 14 and 25 first-level industries, and in every case the nominal “dominant” sector accounts for only 15–21% of members (Community 8: 25 industries, 15% light manufacturing; Community 20: 16 industries, 20% basic chemicals; Community 12: 18 industries, 20% household appliances; Community 5: 14 industries, 21% telecom). Yet the force-directed backbones remain coherent: a dense, strongly weighted core of cross-industry stocks anchors each community, with looser industry-specific spokes radiating outward. The co-movement that binds these constituents is therefore difficult to explain solely as a restatement of industry membership. Instead, the structure is consistent with cross-sector shared return dynamics: where an industry happens to dominate a co-movement bloc the partition can recover it, but many communities are mixed assemblies whose topology is not reducible to Shenwan labels alone.

## 5. Discussion

The observed level decay can be interpreted alongside the rank agreement between bands: different wavelet bands order stock pairs similarly, consistent with each scale partly re-expressing a shared dependence structure. Clustering the same bands independently nonetheless yields distinct partitions. Cross-frequency dependence overlap in this sense is a statement about information *shared between frequency bands*: the bands overlap in what they encode about pairwise dependence, so a band adds less than an independent view would. The overlap is measurable but incomplete, and this conclusion rests on the measures jointly rather than on any one of them: rank agreement is substantial (mean ρ = 0.769), cross-band NMI is low under a fixed binning (0.123), and independent clustering of the bands does not reproduce a common partition (mean pairwise ARI = 0.328). The bands are therefore not interchangeable. It is therefore a different quantity from the directed information *flow between communities* that transfer-entropy analyses of financial networks measure [29]: that literature asks how a shock in one sector propagates to another, whereas the overlap documented here concerns how much the frequency views of a single dependence structure duplicate one another. The two are complementary—inter-community information transfer at a given horizon is one mechanism by which a common backbone could come to be expressed across several bands at once. The value of the large grid is therefore not a novelty claim by scale alone, but the demonstration that the level ordering—the observed scale near-invariance—is stable across many reasonable modeling choices rather than being driven by a single selected configuration.

A common-factor interpretation is consistent with this pattern, but it should be read as a structural interpretation rather than as an identified factor model. In the A-share market, macroeconomic shocks, industry-wide news, liquidity conditions, and style factors can affect returns across multiple investment horizons, consistent with documented multi-factor structures in Chinese equity markets [35]. If such drivers impose a shared dependence backbone, then different wavelet bands should rank stock-pair similarities in a similar way. The high cross-frequency rank agreement and the weak sensitivity to the layer-distance weight are compatible with this possibility. The factor controls provide further evidence: mean cross-band rank agreement falls from 0.769 to 0.677 after removing a leave-one-out market factor and to 0.486 after additionally removing within-industry means, and the information-based measure falls proportionally further, retaining 74% and 34% of its raw value. These projections reduce the measured overlap, with a larger proportional reduction in the information-based measure than in rank agreement, while a substantial share of the rank agreement survives both projections. Because only a market factor and static industry means are projected out—not style, liquidity, or size exposures—these figures describe sensitivity to the two specified projections rather than establish the contribution of common factors in general. The industry step carries a further limit: it removes means within the 106 Tushare groups while ARI is evaluated against the 31 Shenwan sectors, and because the two classifications overlap the projection can remove part of what the evaluation then measures. The cross-industry communities in Figure 7 point in the same direction: they contain coherent co-movement cores that are not reducible to a single Shenwan industry, indicating that the recovered topology is not merely a restatement of static industry labels.

Both design choices can be discussed in light of this overlap. The relative success of shallow decompositions is a property of the fused representation rather than evidence that any isolated band is uniquely responsible: deeper levels recursively split the low-frequency approximation and add the resulting bands to the fused score, but the additional bands may partly overlap in their pair-ranking information and may alter the dependence structure emphasized by the fused representation. Changing *J* also changes the set of bands entering the fusion operator, so this account is an interpretation of the observed pattern and not a property isolated from the fusion rule. The results also show that the more elaborate distance-weighting scheme delivers no stable gain over the simplest fusion rule. Shallow decomposition with equal-weight fusion is therefore a reasonable default within this pipeline and this market, and denoising is one possible interpretation of this pattern, although it is not directly tested here. We report this as a reading compatible with the evidence and not as an established mechanism: an explicit noise model, a reconstruction-error analysis, a comparison against an alternative denoising procedure, and out-of-sample validation would be required to identify denoising as the cause, and none is undertaken here.

The dependence overlap also bears on how such networks are used downstream. Recent dynamic stock-network evidence shows that community evolution, node migration, and network modularity can be translated into market-stability and sector-rotation diagnostics [11]; our results speak to the frequency dimension of such constructions. Because the bands rank stock pairs similarly, treating them as fully independent information sources is not warranted here, and interpreting every frequency layer separately adds little beyond the shallow, equal-weight fused network. The band-wise partitions are nonetheless not identical, so this is a statement about diminishing returns rather than about interchangeable bands.

## 6. Conclusions

For the Chinese A-share market, wavelet frequency bands show substantial agreement in stock-pair dependence rankings and yield distinct community partitions, with a mean pairwise ARI of 0.328 under independent band clustering. Mean cross-band NMI is 0.123 and falls further after removing a market factor and static industry means. These results indicate incomplete cross-frequency dependence overlap. Within the tested pipeline, industry alignment declines with increasing decomposition depth, but this comparison does not isolate the contribution of individual frequency bands or establish the mechanism underlying the decline. Two practical consequences follow. Within this pipeline, shallow decomposition with equal-weight frequency fusion is a simpler and more robust default than deep decomposition with distance-weighted fusion. And ecology-derived frameworks such as WaveClust, whose design premise is genuine cross-scale heterogeneity, require empirical validation before application to financial returns, since that premise is not strongly supported here.

Whether the cross-frequency dependence overlap documented here extends to other markets and asset classes remains an open empirical question. The diagnostics used in this study may provide a useful starting point for investigating this question. These include band-wise rank agreement, an information-based measure, independent band clustering, and factor controls.

## Figures and Tables

**Table 1:** Time windows and eligible stock counts.

Window	Days	Stocks
2024–2025	485	4612
2023–2025	727	4207
2021–2025	1212	3425
2019–2025	1699	2773

**Table 2:** Best clustering performance by decomposition level (2019–2025).

Level	Best ARI	Communities	Largest
2	0.3809	25	220
3	0.3402	25	222
4	0.2815	35	189
5	0.2247	31	213
6	0.1385	35	163

## Data Availability

The data used in this study are available at https://huggingface.co/datasets/Chream/a-share-finance-data (accessed on 6 September 2026). Access is granted upon application and approval by the repository owner. The analysis code is openly available at https://github.com/DreamEnding/WaveClust_Analysis (accessed on 6 September 2026).

## References

[1] Lo A.W. (2004). The Adaptive Markets Hypothesis: Market Efficiency from an Evolutionary Perspective. J. Portf. Manag..

[2] Arthur W.B. (1999). Complexity and the Economy. Science.

[3] Mantegna R.N., Stanley H.E. (1999). An Introduction to Econophysics: Correlations and Complexity in Finance.

[4] Mantegna R.N. (1999). Hierarchical Structure in Financial Markets. Eur. Phys. J. B Condens. Matter Complex Syst..

[5] Tumminello M., Aste T., Di Matteo T., Mantegna R.N. (2005). A Tool for Filtering Information in Complex Systems. Proc. Natl. Acad. Sci. USA.

[6] Onnela J.P., Chakraborti A., Kaski K., Kertész J., Kanto A. (2003). Dynamics of Market Correlations: Taxonomy and Portfolio Analysis. Phys. Rev. E.

[7] Wen F., Yang X., Zhou W.X. (2019). Tail Dependence Networks of Global Stock Markets. Int. J. Finance Econ..

[8] Wu S., Tuo M., Xiong D. (2015). Network Structure Detection and Analysis of Shanghai Stock Market. J. Ind. Eng. Manag..

[9] Tan L., Zhang X., Zhang X. (2024). Retail and Institutional Investor Trading Behaviors: Evidence from China. Annu. Rev. Financ. Econ..

[10] Qi B. (2023). Effectiveness of price limits: Evidence from China’s ChiNext market. PLoS ONE.

[11] Huang Z., Tang M., Zhou Y. (2026). Dynamic Market Structure and Stock Behavior Revealed by Stock Correlation Networks. Phys. A Stat. Mech. Its Appl..

[12] Dacorogna M., Müller U., Olsen R., Pictet O. (2001). Defining Efficiency in Heterogeneous Markets. Quant. Finance.

[13] Müller U.A., Dacorogna M.M., Davé R.D., Pictet O.V., Olsen R.B., Ward J.R. (1995). Fractals and Intrinsic Time—A Challenge to Econometricians.

[14] Baruník J., Křehlík T. (2018). Measuring the Frequency Dynamics of Financial Connectedness and Systemic Risk. J. Financ. Econom..

[15] Mallat S.G. (1989). A Theory for Multiresolution Signal Decomposition: The Wavelet Representation. IEEE Trans. Pattern Anal. Mach. Intell..

[16] Percival D.B., Walden A.T. (2000). Wavelet Methods for Time Series Analysis.

[17] Wang G.J., Xie C., Chen S. (2017). Multiscale Correlation Networks Analysis of the U.S. Stock Market: A Wavelet Analysis. J. Econ. Interact. Coord..

[18] Rua A., Nunes L.C. (2012). A Wavelet-Based Assessment of Market Risk: The Emerging Markets Case. Q. Rev. Econ. Finance.

[19] Gençay R., Gradojevic N., Selçuk F., Whitcher B. (2010). Asymmetry of Information Flow Between Volatilities Across Time Scales. Quant. Finance.

[20] Li J., Shi Z., Li X. (2006). Genetic Programming with Wavelet-Based Indicators for Financial Forecasting. Trans. Inst. Meas. Control.

[21] Lahmiri S. (2012). A Clustering Approach to Examine the Dynamics of the NASDAQ Topology in Times of Crisis. Manag. Sci. Lett..

[22] Martin-Platero A.M., Cleary B., Kauffman K., Preheim S.P., McGillicuddy D.J., Alm E.J., Polz M.F. (2018). High Resolution Time Series Reveals Cohesive but Short-Lived Communities in Coastal Plankton. Nat. Commun..

[23] Plerou V., Gopikrishnan P., Rosenow B., Amaral L.A.N., Guhr T., Stanley H.E. (2002). Random Matrix Approach to Cross Correlations in Financial Data. Phys. Rev. E.

[24] Eom C., Park J.W. (2017). Effects of Common Factors on Stock Correlation Networks and Portfolio Diversification. Int. Rev. Financ. Anal..

[25] Chordia T., Roll R., Subrahmanyam A. (2000). Commonality in Liquidity. J. Financ. Econ..

[26] Hasbrouck J., Seppi D.J. (2001). Common Factors in Prices, Order Flows, and Liquidity. J. Financ. Econ..

[27] Jiang X.F., Chen T.T., Zheng B. (2014). Structure of Local Interactions in Complex Financial Dynamics. Sci. Rep..

[28] MacMahon M., Garlaschelli D. (2015). Community Detection for Correlation Matrices. Phys. Rev. X.

[29] Korbel J., Jiang X., Zheng B. (2019). Transfer Entropy between Communities in Complex Financial Networks. Entropy.

[30] Tushare (2026). Tushare Pro Financial Data API. https://tushare.pro.

[31] Lee G.R., Gommers R., Waselewski F., Wohlfahrt K., O’Leary A. (2019). PyWavelets: A Python Package for Wavelet Analysis. J. Open Source Softw..

[32] Kunegis J., Schmidt S., Lommatzsch A., Lerner J., De Luca E.W., Albayrak S. Spectral Analysis of Signed Graphs for Clustering, Prediction and Visualization. Proceedings of the 2010 SIAM International Conference on Data Mining.

[33] Ng A.Y., Jordan M.I., Weiss Y. On Spectral Clustering: Analysis and an Algorithm. Proceedings of the Advances in Neural Information Processing Systems.

[34] Hubert L., Arabie P. (1985). Comparing Partitions. J. Classif..

[35] Liu J., Stambaugh R.F., Yuan Y. (2019). Size and Value in China. J. Financ. Econ..

